# Optimizing line blot assays: impact of band intensity adjustment on diagnostic performance in myositis autoantibodies testing

**DOI:** 10.1007/s10067-026-08153-4

**Published:** 2026-05-27

**Authors:** Francisco Javier Hüttmann, Albert Pérez-Isidro, María Ortiz-Fernández, María Torradeflot, Noemí de Moner, Fernanda Hernández-González, Estíbaliz Ruiz-Ortiz, Sergio Prieto-González

**Affiliations:** 1 Department of Autoimmune Diseases, Hospital Clínic, Barcelona, Spain; 2 Department of Immunology, Centre Diagnòstic Biomèdic, Hospital Clínic de Barcelona, Barcelona, Spain; 3 Institut d’Investigacions Biomèdiques August Pi I Sunyer (IDIBAPS), Barcelona, Spain; 4 Department of Respiratory Medicine, Hospital Clínic de Barcelona, Barcelona, Spain; 5 Department of Internal Medicine, Hospital Clínic de Barcelona, Barcelona, Spain; 6 University of Barcelona, Barcelona, Spain

**Keywords:** Autoantibody testing, Band intensity adjustment, Diagnostic performance, Idiopathic inflammatory myopathies, Line blot assay, Myositis-specific autoantibodies

## Abstract

**Introduction:**

Line blot assays (LBA) have several limitations regarding diagnostic validity, as sensitivity and specificity vary considerably among antigens.

**Objective:**

The aim of this study was to assess whether the adjustment of each specific band intensity (BI) relative to the positive control band (PCB), combined with increasing the cut-off value from 15 to 20, improves the diagnostic performance of the LBA for detecting autoantibodies related to idiopathic inflammatory myopathies in our patient cohort.

**Methods:**

We included all serum samples tested for myositis‑specific antibodies (MSA) and myositis‑associated antibodies (MAA) between January 2022 and April 2023 at the Immunology Department of Hospital Clínic of Barcelona. Two analyses were performed: Analysis A, using the manufacturer’s recommended cut-off (BI ≥ 15), and Analysis B, applying the proposed BI adjustment relative to the PCB and raising the cut-off to 20 in samples that were positive in Analysis A.

**Results:**

A total of 939 patients were included. Using Analysis A, 280/939 (29.8%) patients had at least one positive result, whereas 659/939 (70.2%) were negative for all MSA and MAA. After applying Analysis B, 151/280 (53.9%) remained positive, while 129/280 (46.1%) became negative. With Analysis A, sensitivity was 76%, specificity 75%, positive predictive value (PPV) 24%, and negative predictive value (NPV) 96%. Under Analysis B, sensitivity was 70%, specificity 89%, PPV 41%, and NPV 96%.

**Conclusions:**

Applying the proposed BI adjustment effectively removes weakly positive results with low specificity in an objective and reproducible manner, thereby increasing the overall specificity of the LBA.

**Table:** 

**Key Points** • *Applying the proposed band intensity removes weakly positive results*. • *This approach enhances the specificity, and positive predictive value of a commercial line blot assay*.

**Supplementary Information:**

The online version contains supplementary material available at 10.1007/s10067-026-08153-4.

## Introduction

Idiopathic inflammatory myopathies (IIM) are a heterogeneous group of systemic autoimmune disorders, historically defined by muscle inflammation and proximal muscle weakness. However, it is now widely recognized that IIM frequently involves multiple organs, including joints, skin, and lungs, particularly in the form of interstitial lung disease (ILD). Furthermore, certain clinical subtypes may present without overt muscle involvement (amyopathic), emphasizing the diversity and complexity of these conditions [1, 2]. The IIM spectrum includes dermatomyositis (DM), polymyositis (PM), immune-mediated necrotising myopathy (IMNM), sporadic inclusion body myositis (sIBM), and anti-synthetase syndrome (ASS) [3]. Moreover, myositis may also occur as a manifestation of other autoimmune systemic diseases, or as a part of an overlap syndrome.

Classification of IIM has evolved substantially due to advances in immunological techniques, which have enabled the identification of numerous myositis-specific autoantibodies (MSA) and myositis‑associated autoantibodies (MAA) [4]. Different MSAs have been identified, including anti-aminoacyl-tRNA synthetases (ARS), anti-Mi2, anti-TIF1γ, anti-SAE, anti-NXP2, anti-MDA5, anti-SRP, and anti-HMGCR. These autoantibodies are detected in approximately 60–70% of patients with IIM and play a key role in defining clinically meaningful subgroups. MAAs, while less specific, appear not only in IIM but also in other systemic autoimmune diseases, and overlap syndromes [5]. The detection of these autoantibodies has become essential for diagnosis, subclassification and prognostic assessment [6, 7].

Among available laboratory methods, immunoprecipitation (IP) remains the gold standard for detecting MSAs because it preserves native antigen conformation and offers high specificity. Nevertheless, its use is restricted to a limited number of specialized laboratories due to its technical complexity and labor‑intensive procedures [8]. Consequently, commercial line blot (LBA) and dot blot assay using recombinant antigens have become widely adopted in clinical practice [9]. These assays offer several advantages: rapid processing, multiplex antigen detection, automated interpretation, and semi‑quantitative output [10]. However, significant variability in diagnostic performance has been reported due to differences in antigen quality, recombinant constructs, and analytical thresholds [11, 12]. Recent work has highlighted the need to improve the analytical performance of EuroLineBlot, one of the most commonly used LBA. Chang et al. (2023) proposed a standardized correction method that adjusts each antigen’s band intensity (BI) relative to the positive control band (PCB) included in each strip. Their findings demonstrated that using PCB‑adjusted BI values —combined with a stricter positivity threshold of ≥ 20 (rather than 15)— significantly improve the diagnostic performance of this LBA. Moreover, this adjustment reduced the rate of false-positive results in healthy controls, further supporting its usefulness in clinical practice [13].

The implementation of PCB-adjusted BI values together with a higher cut-off, represents a key step toward the standardization of this immunoassay and could optimize serological interpretation in patients with suspected IIM. In this context, our study aims to evaluate whether implementing the same strategy, enhances the diagnostic performance of this LBA for detecting MSAs and MAAs in a large, real‑world cohort assessed for suspected IIM at a tertiary care center.

## Patients & methods

### Samples

**Fig.1:**
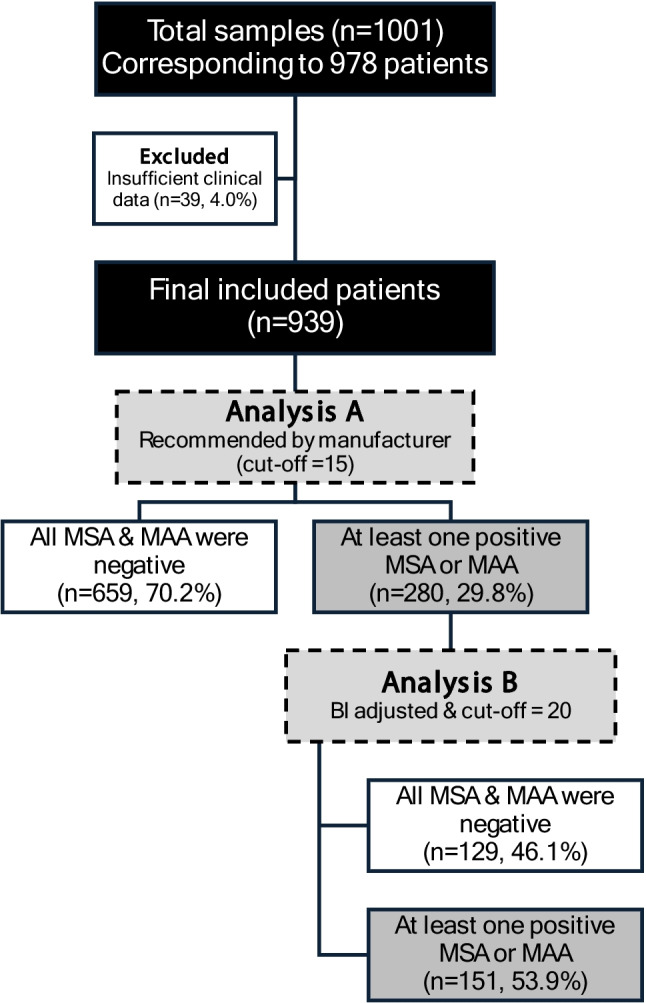
Workflow of the study. Abbreviations: MSA: Myositis‑specific autoantibodies, MAA: Myositis‑associated autoantibodies, BI: Band intensity

This study was designed as an observational, descriptive–analytical, retrospective investigation conducted at Hospital Clínic de Barcelona. All serum samples for which MSA and MAA were tested as part of routine clinical care between January 2022 and April 2023 were included. A total of 1001 serum samples corresponding to 978 individual patients were identified. Patients for whom insufficient clinical data were available to establish a final diagnosis were excluded from the analysis (*n* = 39, 4.0%), resulting in a final cohort of 939 patients (Fig. 1).

### Patients’ diagnosis

To establish the final clinical diagnosis, a comprehensive retrospective review of electronic medical records was conducted. Patients were classified according to the 2017 EULAR/ACR classification criteria for adult IIM [14]. Additional classification systems were applied for specific subtypes: the CLASS project criteria for ASS, the 2016 ENMC clinic‑sero‑pathologic criteria for IMNM, and the 2013 ENMC diagnostic criteria for IBM [15]. Patients with other systemic autoimmune diseases presenting with inflammatory muscle involvement were grouped as having secondary myositis.

### Myositis autoantibodies detection

All included serum samples were processed using commercial LBA (EUROIMMUN, Lübeck, Germany) on the EuroBlotOne automated platform according to the manufacturer’s instructions. Three different panels were used depending on clinical request and availability: EUROLINE Myositis Antigen Profile 3 IgG (Mi-2β, Ku, PM-Scl100, PM-Scl75, Jo1, SRP, PL7, PL12, EJ, OJ and Ro52), Autoimmune Inflammatory Myopathies 16 Ag IgG (Mi-2α, Mi-2β, TIF1γ, MDA5, NXP2, SAE1, Ku, PM-Scl100, PM-Scl75, Jo1, SRP, PL7, PL12, EJ, OJ and Ro52), and Autoimmune Inflammatory Myopathies 16 Ag cN‑1A IgG (Mi-2 alpha, Mi-2 beta, TIF1γ, MDA5, NXP2, SAE1, Ku, PM-Scl100, PM-Scl75, Jo1, SRP, PL7, PL12, EJ, OJ, Ro52, and cN-1A). Each strip contains multiple recombinant antigens immobilized in discrete lines. The BI of each antigen was quantified using the EUROLineScan software provided by the manufacturer. According to assay specifications, a PCB is incorporated into each strip; adequate performance of the test requires a BI of the PCB > 50. In our laboratory, based on local experience, assays are considered valid only when the BI of the PCB is > 100. Once PCB validity was confirmed, the BI of each antigen was interpreted according to the manufacturer’s semi‑quantitative categories: 0–15 negative, 16–35 weakly positive, 36–70 moderately positive, and ≥ 71 strongly positive.

To evaluate whether analytical adjustment improves diagnostic performance, two complementary analyses were performed. In Analysis A, results were interpreted strictly using the manufacturer’s standard cut‑off, considering BI ≥ 15 as positive. In Analysis B, positivity was reassessed by applying a correction formula proposed by Chang et al. (2023), whereby the BI of each antigen was divided by the BI of the corresponding PCB and multiplied by 100, generating an adjusted BI (aBI) value. A stricter positivity threshold of ≥ 20 was then applied to the aBI. This second analysis was performed only in patients with at least one positive result in Analysis A (Fig. 1). Under our analytical conditions, it is mathematically impossible for a negative result in Analysis A to become positive after applying the adjustment formula proposed by Chang et al. (Analysis B).

In a subset of patients included in Analysis B, indirect immunofluorescence (IIF) on HEp‑2 cells was reviewed. IIF was performed using standard laboratory protocols. IIF staining at titre ≥ 1:80 was considered positive. Patterns were identified according to the International Consensus on ANA patterns (ICAP) [16]. The IIF results were compared between patients who remained positive after BI adjustment and those who became negative.

### Statistical analysis

Descriptive statistics were used to summarize demographic, serological, and clinical data. Sensitivity, specificity, positive predictive value (PPV), and negative predictive value (NPV) were calculated from 2 × 2 contingency tables for Analysis A and Analysis B using the final clinical diagnosis as the reference standard. All analyses were performed using IBM SPSS Statistics software (version 29.0; IBM Corp., Armonk, NY, USA). Ninety-five percent confidence intervals were estimated using exact binomial methods. Paired categorical data were compared using McNemar’s test. Although the comparison is not fully paired across the entire cohort, McNemar’s test was applied within the subset of patients with initial positive results to explore the impact of the adjustment. A p value < 0.05 was considered statistically significant.

## Results

A total of 39 patients were excluded from the study, resulting in a final study population of 939 patients. Clinical data, laboratory results, and other complementary tests from the 939 patients were reviewed. Based on these data, and according to the classification criteria for each disorder, patients were classified as having a diagnosis of IIM (*n* = 89, 9.5%) or not having a diagnosis of IIM (*n* = 850, 90.5%). Demographic characteristics and clinical manifestations of all patients included in the study are shown in Table 1. Samples originated from 21 different clinical departments, being the most frequent Pulmonology (39.0%), Internal Medicine (23.0%), and Autoimmune Diseases (13.5%). All samples showed a PCB intensity greater than 100, confirming correct assay performance.

**Table 1:** Demographic characteristics and clinical manifestations of patients

Feature	IIM *n* = 89 *n* (%)	No IIM *n* = 850 *n* (%)	*p* value
Age mean years (Min–Max)	54 (5–86)	62 (35—82)	< 0.001
Female (%)	46 (52)	433 (51)	0.894
Muscle weakness (%)	68 (75)	195 (23)	< 0.001
Skin involvement (%)	48 (54)	59 (7)	< 0.001
Elevated muscle enzymes (%)	68 (77)	25 (3)	< 0.001
Myopathic EMG pattern (%)	20 (22)	17 (2)	< 0.001
ILD (%)	22 (25)	527 (62)	< 0.001
Arthritis/arthralgia (%)	7 (8)	17 (2)	0.005
Raynaud (%)	13 (15)	178 (21)	0.158
Associated cancer (%)	6 (7)	17 (2)	0.016

*IIM* idiopathic inflammatory myopathies, *ILD* interstitial lung disease, *EMG* electromyography

Using the manufacturer’s standard interpretation (Analysis A), 280 of 939 patients (29.8%) had at least one positive autoantibody result, while 659 patients (70.2%) tested negative for all autoantibodies included in the LBA. After applying the adjusted BI calculation and stricter cut-off (Analysis B), 151 of the 280 initially positive patients (53.9%) remained positive, whereas 129 patients (46.1%) converted to negative (Fig. 1; Suppl Table 1).

The prevalence of each autoantibody under Analyses A and B is detailed in Table 2. Under Analysis A (recommended by the manufacturer), the most frequent MSA was anti‑PL7 (13.5%), followed by anti‑Jo1 (8.2%) and anti‑TIF1γ (6.4%). Among MAAs, anti‑Ro52 was the most common (32.1%). After applying Analysis B (suggested by Chang et al. [13], anti‑Jo1 (5.7%) became the most frequent MSA, followed by anti‑PL7 (4.2%), while anti‑Ro52 remained the predominant MAA (25.3%). More than half (52%) of the initially positive results became negative after adjustment, particularly for PL7, MDA5, Mi2b, and PM/Scl100 antigens.

**Table 2:** Frequency of myositis specific and associated autoantibodies according to analyses A and B

Autoantibodies	Antigen	Number of determinations	Analysis A	Analysis B					
			Positive results *n* (%)	Positive results *n* (%)			Negative results *n* (%)		
			*n* = 371	*n* = 178	IIM *n* = 91	no IIM *n* = 87	*n* = 193	IIM *n* = 37	no IIM *n* = 156
Myositis-specific	Jo1	280	23 (8.2)	16 (5.7)	10	6	7 (2.5)	1	6
	EJ	280	4 (1.4)	2 (0.7)	2	0	2 (0.7)	0	2
	OJ	280	1 (0.3)	0 (0.0)	0	0	1 (0.4)	0	1
	PL7	280	38 (13.5)	12 (4.2)	6	6	26 (9.3)	3	23
	PL12	280	8 (2.8)	1 (0.3)	1	0	7 (2.5)	3	4
	MDA5	280	15 (5.3)	5 (1.7)	5	0	10 (3.6)	2	8
	TIF1γ	296	19 (6.4)	8 (2.7)	7	1	11 (3.7)	2	9
	NXP2	271	8 (2.9)	2 (0.7)	2	0	6 (2.2)	1	5
	SAE1	271	13 (4.8)	1 (0.3)	1	0	12 (4.4)	1	11
	Mi2	19	2 (10.5)	0 (0.0)	0	0	2 (10.5)	0	2
	Mi2a	271	16 (5.9)	5 (1.8)	4	1	11 (4.1)	5	6
	Mi2b	252	19 (7.5)	5 (1.9)	2	3	14 (5.6)	3	11
	SRP	271	13 (4.8)	6 (2.2)	4	2	7 (2.6)	3	4
Myositis-associated	Ro52	280	90 (32.1)	71 (25.3)	34	37	19 (6.8)	5	14
	PM/Scl75	280	37(13.2)	14 (5.0)	1	13	23 (8.2)	2	21
	PM/Scl100	280	36 (12.8)	8 (2.8)	1	7	28 (10.0)	3	25
	Ku	280	16 (5.7)	10 (3.5)	4	6	6 (2.1)	2	4
	cN1A	69	13 (18.8)	12 (17.3)	7	5	1 (1.4)	1	0

In patients who underwent Analysis B, IIF patterns on HEp-2 cells were also reviewed. Only 6 of 151 patients (3.9%) who remained positive showed a negative HEp‑2 IIF result, whereas 31 of 129 patients (24.0%) who became negative showed negative HEp‑2 results, supporting the interpretation that many low‑intensity LBA results lack clinical correlation. Using the manufacturer’s cut‑off (Analysis A), diagnostic performance was as follows: sensitivity 76.4%, specificity 75.1%, PPV 24.3%, and NPV 96.8% (Table 2). After adjustment (Analysis B), sensitivity was 70.8%, specificity increased to 89.6%, PPV improved to 41.7%, and NPV remained 96.7%. Comparative performance between both analyses is summarized in Table 3. A total of 89 of 939 patients (9.1%) were diagnosed with IIM. The distribution of classification subtypes was DM 39/89 (43.8%), ASS 20/89 (22.5%), IMNM 15/89 (16.8%), PM 4/89 (4.5%), and other myositis forms 11/89 (12.4.%). Their clinical and demographic features, according to both analyses, are summarized in Table 4. Among the 659 patients with negative results in Analysis A, 21 (3.1%) were clinically diagnosed with IIM. The most frequent subtypes in this seronegative group were DM 9/21 (42.8%), IMNM 8/21 (38.2%). Among these patients, 5/8 were anti-HMGCR positive and 3/8 negative. The other subtypes were PM 1/21 (4.7%), IBM 1/21 (4.7%) and secondary autoimmune associated myopathy 2/21 (9.5%).

**Table 3:** Diagnostic performance comparison between Analysis A and Analysis B

Diagnostic test characteristics	Analysis A % (95% confident intervals)	Analysis B % (95% confident intervals)
Sensitivity	76.4% (66.61–84.02)	70.8% (60.64–79.22)
Specificity	75.1% (72.04–77.85)	89.6% (87.42–91.52)
PPV	24.3% (19.63–29.63)	41.7% (34.16–49.70)
NPV	96.8% (95.18–97.91)	96.7% (95.21–97.74)

*NPV* Negative predictive value, *PPV* Positive predictive value

**Table 4:** Clinical characteristics of patients with IIM

	DM *n* = 39 (43.8%)	ASS *n* = 20 (22.5%)	IMNM *n* = 15 (16.8%)	PM *n* = 4 (4.5%)	Others *n* = 11 (12.4%)
Female sex (%)	26 (66.7)	9 (45.0)	11 (73.0)	4 (100)	10 (91.0)
Type of involvement					
Cutaneous (%)	35 (89.7)	11 (55.0)	1 (6.6)	0 (0.0)	1 (9.1)
Muscle (%)	32 (82.0)	11 (55.0)	14 (93.3)	3 (75.0)	8 (72.7)
EMG (%)	10 (25.6)	1 (5.0)	8 (53.3)	0 (0.0)	0 (0.0)
Compatible biopsy (%)	17 (43.6)	4 (20.0)	11 (73.3)	1 (25.5)	4 (36.4)
Raynaud (%)	12 (30.7)	9 (45.0)	0 (0.0)	0 (0.0)	2 (18.2)
Arthritis (%)	4 (10.2)	8 (40.0)	0 (0.0)	0 (0.0)	2 (25.0)
ILD (%)	6 (15.4)	18 (90.0)	1 (6.6)	0 (0.0)	0 (0.0)
Cancer (%)	4 (10.3)	2 (10.0)	1 (6.6)	0 (0.0)	1 (6.2)

*ASS* Antisynthetase syndrome, *DM* Dermatomyositis, *IMNM* Immune mediated necrotizing myopathy, *PM* Polymyositis, *Others*: Inclusion body myositis, secondary myositis, *EMG* electromyography, *ILD* interstitial lung disease, *IIM* idiopathic inflammatory myopathies

## Discussion

The present study evaluated the diagnostic performance obtained by adjusting the BI of each autoantibody in relation to the BI of the PCB, together with increasing the cut-off value of the LBA assay from 15 to 20 for the detection of MSA and MAA in a cohort of patients with suspected IIM.

Our findings show that, when applying the manufacturer’s proposed cut-off in our cohort, the LBA achieves good sensitivity and NPV but has limitations in specificity —particularly a low PPV— which may lead to false-positive classifications.

After applying the adjustment proposed by Chang et al. in 2023, an improvement was observed in specificity (89.6% vs 75.1%) and in PPV (41.7% vs 24.3%), with a moderate decrease in sensitivity (76.4% vs 70.8%). These findings support the usefulness of employing this proposal with a higher cut-off as a strategy to reduce the rate of false-positive results, thereby improving the diagnostic accuracy of this method for the evaluation of MSAs and MAAs.

These results are consistent with those reported in previous work. In 2021, Beaton et al. analyzed the performance of LBA in cohorts of patients with suspected IIM and observed that using a higher cut-off value (intensity > 1 +) improved the correlation between LBA results and definitive IIM diagnoses [17]. In another study, the manufacturer’s recommended cut-off was compared with values derived from the 99th percentile of healthy controls. Applying these thresholds reduced the prevalence of MSA positivity among controls and increased specificity [12]. Mecoli et al. also observed that using lower cut-off values was associated with an increased detection of positive results, without clear clinical correlation, particularly in antigens such as PL-7 and PL-12 [18]. The consistency of our findings with those studies reinforces the validity of using both adjusted BI and stricter thresholds to enhance diagnostic accuracy.

We also evaluated the prevalence of each autoantibody using both cut-off values. After modifying the cut-off (Analysis B), more than 50% of the autoantibodies results became negative, mainly those targeting PL-7, MDA5, Mi-2β, and PM/Scl-100. This finding suggests that commercial kits may detect low-affinity autoantibodies or weak, non-specific interactions at low intensities, leading to false-positive results that are resolved when applying a stricter threshold. In this regard, several factors may account for these observations. First, some recombinant antigens used in commercial LBA may be particularly prone to non specific or low affinity binding. Second, certain autoantibodies are known to have lower disease specificity and may be detected at low levels in non IIM conditions. Finally, weak signal intensities close to the manufacturer’s cut off may reflect background reactivity rather than clinically meaningful autoimmunity. Similar changes were observed for other antigens, such as Mi2 and SAE1, whose frequencies decreased markedly after adjustment, further supporting the notion that reactivities detected at the original cut-off may represent low-intensity or clinically irrelevant findings, consistent with previously published literature [19].

In our study, IIF results on HEp-2 cells provided additional support for this interpretation. Patients who remained positive after BI adjustment rarely exhibited negative IIF patterns, whereas negative IIF was notably more common among those whose LBA results became negative after adjustment. This reinforces the conclusion that many low‑intensity LBA results reflect either background reactivity or clinically insignificant findings.

It is important to highlight that five patients with confirmed IIM diagnoses became seronegative after the BI adjustment (Supplementary Table 2). This does not invalidate the analytic correction; rather, it underscores the principle that serological testing must always complement—rather than replace—clinical assessment. Autoantibody negativity does not exclude IIM, as observed in certain DM phenotypes. Clinical and histopathological data remain central to diagnosis. Additionally, it is important to note that anti-HMGCR autoantibodies are not included in the LBA panel and must be detected using separate assays.

This study has certain limitations. First, the analyses were performed using a specific commercial panel; therefore, generalization of the results to other platforms or manufacturers may not be appropriate. Second, the retrospective study design implies that data analysis was based on pre-existing information, which may include variability in the clinical and serological data available. A total of 39 patients were excluded from the study. Overall, the clinical context in which autoantibody testing was requested in these patients was comparable to that of the included cohort, predominantly involving suspicion of systemic autoimmune disease, ILD, or possible inflammatory muscle involvement. However, due to insufficient clinical data, a definitive diagnosis could not be established in these patients, resulting in a final study population of 939 patients. Clinicians had access to autoantibody results when classifying patients, which may introduce incorporation bias; however, we sought to minimize this by integrating autoantibody profiles only as one element within a broader diagnostic framework that included biochemical parameters, muscle biopsy findings, imaging studies, and other clinical data. Third, although IP remains the gold‑standard method for the detection of MSAs, IP data were not available for most of the cases included in our study and were only accessible for a single patient. Finally, incomplete follow‑up or missing diagnostic information resulted in the exclusion of a small proportion of patients.

Despite these limitations, our findings contribute to the growing evidence supporting the need for refined interpretive criteria in commercial LBA. The implementation of PCB‑adjusted BI values and stricter positivity thresholds enhances the clinical utility of these tests and reduces the likelihood of false‑positive interpretations.

## Conclusion

The application of band intensity adjustment relative to the positive control band, combined with increasing the positivity cut‑off from 15 to 20, allows for the elimination of weak, low‑specificity reactions in a reproducible and objective manner. This approach significantly enhances the specificity of the EuroLineBlot assay while maintaining a high negative predictive value. Adopting these adjusted thresholds may enhance diagnostic accuracy in clinical practice when evaluating patients with suspected idiopathic inflammatory myopathies. Further studies, including validation in prospective cohorts, are needed to confirm these findings and assess their applicability in routine practice.

## Supplementary Information

Below is the link to the electronic supplementary material.

Supplementary file1 (DOCX 8.15 KB)

## Data Availability

The datasets generated and/or analysed during the current study are available from the corresponding author on reasonable request.
