# Supplementary material for: Optimizing line blot assays: impact of band intensity adjustment on diagnostic performance in myositis autoantibodies testing
Source: Clin Rheumatol. 2026 May 27;45(7):4391–7. doi: 10.1007/s10067-026-08153-4 (PMC13341838; doi:10.1007/s10067-026-08153-4)
Supplement: Supplementary file 1 — Supplementary file1 (DOCX 8.15 KB) [file 10067_2026_8153_MOESM1_ESM.docx]

Supplementary Table 1.

| Test Mc Nemar (p<0.0001) | Analysis B, positive results (n=151) | Analysis B, negative results (n=788) |
| --- | --- | --- |
| Analysis A, positive results (n= 280) | 151 | 129 |
| Analysis A, negative results (n=659) | 0 | 659 |

Supplementary Table 2. Clinical features of patients with idiopathic inflammatory myopathies who showed MSA negativization after Analysis B.

|  | LBA ANALYSIS A | LBA ANALYSIS B | BIOPSY | MYOPATHIC EMG PATTERN | SKIN INVOLVEMENT |
| --- | --- | --- | --- | --- | --- |
| Patient 1 | TIF1γ + | TIF1γ - | Not performed | Positive | Compatible |
| Patient 2 | PL12+ MDA5+ SRP+ | PL12- MDA5- SRP- | Positive | Positive | Non-specific |
| Patient 3 | Jo1+ PL7+ | Jo1- PL7- | Positive | Positive | Compatible |
| Patient 4 | Ro52+ | Ro52- | Positive | Positive | Compatible |
| Patient 5 | Mi2a+ Mi2b+ | Mi2a- Mi2b- | Positive | Not performed | Non-specific |
